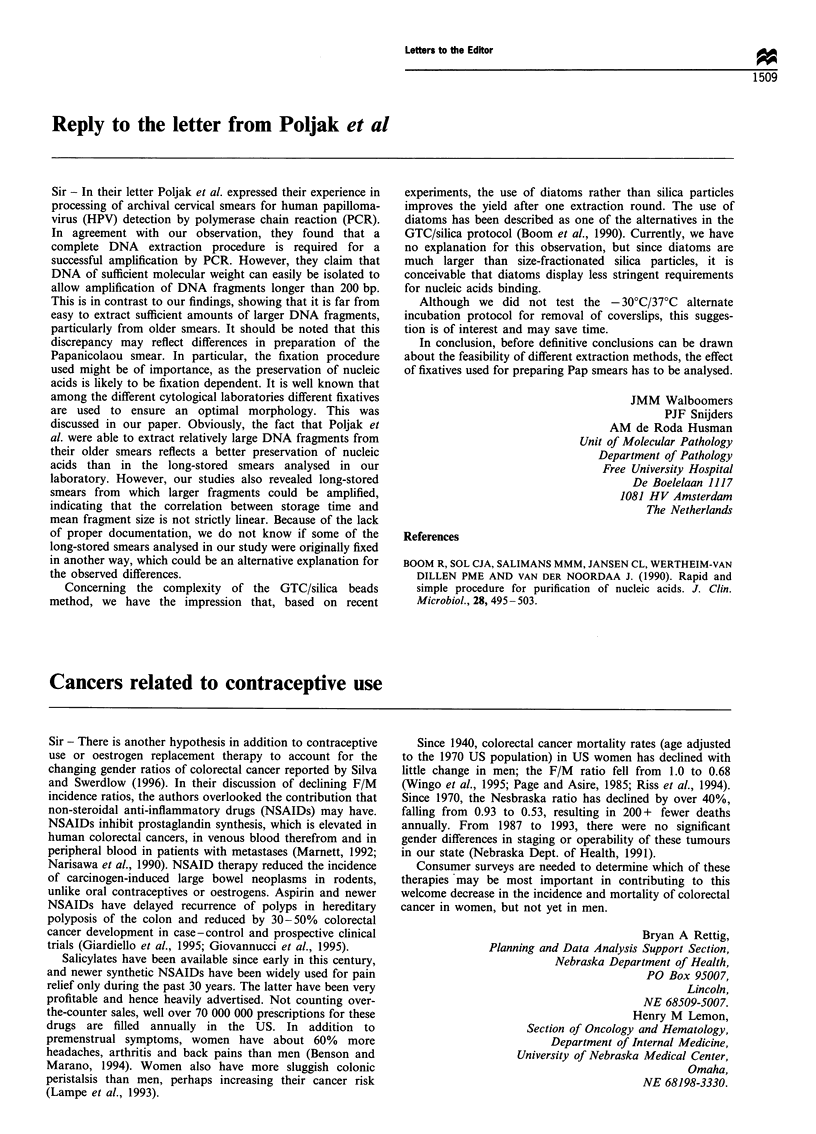# Reply to the letter from Poljak et al

**Published:** 1996-11

**Authors:** JMM Walboomers, PJF Snijders, AM de Roda Husman


### Reply to the letter from Poljak *et al*

Sir – In their letter Poljak *et al.* expressed their experience in processing of archival cervical smears for human papillomavirus (HPV) detection by polymerase chain reaction (PCR). In agreement with our observation, they found that a complete DNA extraction procedure is required for a successful amplification by PCR. However, they claim that DNA of sufficient molecular weight can easily be isolated to allow amplification of DNA fragments longer than 200 bp. This is in contrast to our findings, showing that it is far from easy to extract sufficient amounts of larger DNA fragments, particularly from older smears. It should be noted that this discrepancy may reflect differences in preparation of the Papanicolaou smear. In particular, the fixation procedure used might be of importance, as the preservation of nucleic acids is likely to be fixation dependent. It is well known that among the different cytological laboratories different fixatives are used to ensure an optimal morphology. This was discussed in our paper. Obviously, the fact that Poljak *et al.* were able to extract relatively large DNA fragments from their older smears reflects a better preservation of nucleic acids than in the long-stored smears analysed in our laboratory. However, our studies also revealed long-stored smears from which larger fragments could be amplified, indicating that the correlation between storage time and mean fragment size is not strictly linear. Because of the lack of proper documentation, we do not know if some of the long-stored smears analysed in our study were originally fixed in another way, which could be an alternative explanation for the observed differences.

Concerning the complexity of the GTC/silica beads method, we have the impression that, based on recent experiments, the use of diatoms rather than silica particles improves the yield after one extraction round. The use of diatoms has been described as one of the alternatives in the GTC/silica protocol (Boom *et al.*, 1990). Currently, we have no explanation for this observation, but since diatoms are much larger than size-fractionated silica particles, it is conceivable that diatoms display less stringent requirements for nucleic acids binding.

Although we did not test the −30°C/37°C alternate incubation protocol for removal of coverslips, this suggestion is of interest and may save time.

In conclusion, before definitive conclusions can be drawn about the feasibility of different extraction methods, the effect of fixatives used for preparing Pap smears has to be analysed.

JMM Walboomers
PJF Snijders
AM de Roda Husman
*Unit of Molecular Pathology*
*Department of Pathology*
*Free University Hospital*
*De Boelelaan 1117*
*1081 HV Amsterdam*
*The Netherlands*

### References

BOOM R, SOL CJA, SALIMANS MMM, JANSEN CL, WERTHEIM-VAN DILLEN PME AND VAN DER NOORDAA J. (1990). Rapid and simple procedure for purification of nucleic acids. *J. Clin. Microbiol.*, **28**, 495–503.